# Caring for Pregnant Women with Rheumatic Heart Disease: A Qualitative Study of Health Service Provider Perspectives

**DOI:** 10.5334/gh.1086

**Published:** 2021-12-22

**Authors:** Geraldine Vaughan, Angela Dawson, Michael Peek, Jonathan Carapetis, Vicki Wade, Elizabeth Sullivan

**Affiliations:** 1 Central Queensland University, College of Science and Sustainability, Sydney campus, AU; 2 University of Technology Sydney, Faculty of Health, Sydney, AU; 3 The Australian National University, College of Health and Medicine, Canberra, AU; 4 Telethon Kids Institute, University of Western Australia, and Perth Children’s Hospital, Nedlands WA, AU; 5 Menzies School of Health Research, NT, AU; 6 The University of Newcastle, Faculty of Health and Medicine, Newcastle, AU

**Keywords:** Rheumatic Heart Disease, pregnancy, health services

## Abstract

**Background:**

Rheumatic heart disease (RHD) persists in low-middle-income countries and in high-income countries where there are health inequities. RHD in pregnancy (RHD-P) is associated with poorer maternal and perinatal outcomes. Our study examines models of care for women with RHD-P from the perspectives of health care providers.

**Methods:**

A descriptive qualitative study exploring Australian health professionals’ perspectives of care pathways for women with RHD-P. Thematic analysis of semi-structured interviews with nineteen participants from maternal health and other clinical and non-clinical domains related to RHD-P.

**Results:**

A constellation of factors challenged the provision of integrated women-centred care, related to health systems, workforces and culture. Themes that impacted on the provision of quality woman-centred care included conduits of care – helping to break down silos of information, processes and access; ‘layers on layers’ – reflecting the complexity of care issues; and shared understandings – factors that contributed to improved understandings of disease and informed decision-making.

**Conclusions:**

Pregnancy for women with RHD provides an opportunity to strengthen health system responses, improve care pathways and address whole-of-life health. To respond effectively, structural and cultural changes are required including enhanced investment in education and capacity building – particularly in maternal health – to support a better informed and skilled workforce. Aboriginal Mothers and Babies programs provide useful exemplars to guide respectful effective models of care for women with RHD, with relevance for non-Indigenous women in high-risk RHD communities.

For key goals to be met in the context of RHD, maternal health must be better integrated into RHD strategies and RHD better addressed in maternal health.

## Background

Rheumatic heart disease (RHD) persists in low-middle-income countries and amongst populations in high-income countries who experience a disproportionate burden associated with structural deficiencies, health inequities and discrimination, particularly Indigenous peoples [1,2].

RHD is twice as common in women and is associated with poorer perinatal outcomes and compromised care pathways [3,4,5]. In the high-income country of Australia, RHD is found predominantly amongst Aboriginal and Torres Strait Islander peoples, Māori and Pasifika peoples, migrants from resource-poor countries and refugees. While RHD in pregnancy (RHD-P) is overall rare in Australia (one per 2,500 women), it is exponentially higher in remote Aboriginal communities, with the burden of RHD disease significantly higher in remote northern Australia [6]. An estimated 2–3% of pregnant Aboriginal women each year in the Northern Territory have RHD [3,7]. The last decade has seen a groundswell of research and advocacy positioning RHD in the global health arena [8,9,10,11,12,13,14]. Against this backdrop, particular challenges of providing optimal care for women with RHD-P are increasingly evident [3,15,16,17,18,19,20,21,22].

Previous Australian studies have explored the clinical impact of RHD-P [3,7,16,23]. Women’s perspectives of RHD-P highlighted deficits of care, gaps in understandings and challenges of accessing care for remote-dwelling Aboriginal women [15]. A systematic review has described gaps in the reporting of clinical measures for women with RHD-P [24]. However, there is a lack of understanding of care pathways of women with RHD-P from the perspective of health service providers in Australia.

## Methods

### Aim and objectives

This paper reports the findings of a qualitative study that aimed to provide an understanding of the context of providing care for Australian women with RHD-P. Objectives were to identify gaps and facilitators of optimal woman-centred care [25], through examining health professionals’ knowledge, perspectives, and suggested strategies to meet women’s needs more effectively. Insights aimed to inform policy and practice including guidelines for health professionals and advocacy work.

### Study design

This descriptive qualitative study employed thematic analysis of semi-structured interviews to identify emergent themes.

### Study setting and context

Australia’s health system is a complex mix of service providers primarily funded under a universal health insurance scheme from national and jurisdictional governments and the non-government sector, providing care in public and private hospitals, primary health care services [26] (including Aboriginal Community Controlled Health Organizations – led by local Aboriginal communities) and referred medical services.

**Figure 1:**
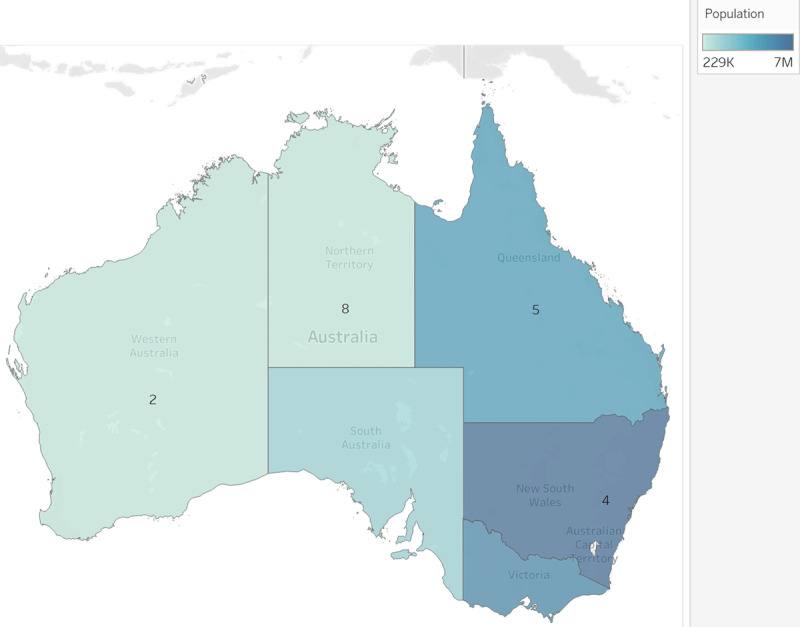
Map of Australia showing jurisdictions, with population and participant distribution.

The interviews were conducted with health care providers predominantly from northern Australia lands (see Figure 1 below) – Northern Territory (NT), northern Western Australia and Far North Queensland – which have the highest prevalence of RHD. These regions are also characterized by their remoteness [27,28], gaps in equitable access to health services and income inequality (non-Indigenous Australians in remote areas reported a median gross personal income 85% higher to that of Indigenous Australians) [29].

There are limited cardiac facilities in these regions (the Northern Territory, with a land area larger than South Africa and over twice that of France, has no cardiothoracic surgery program [30,31], with gaps in the provision of equitable maternity services, particularly for Aboriginal women [32].

### Participant recruitment

Purposive maximum variation sampling based on location, sector and professional role was undertaken to provide meaningful perspectives on aspects of models of care for RHD-P [33]. Individuals were recruited based on experience of providing care for women with RHD-P, and/or their professional RHD work at program/policy levels. Initial contact was followed by a subsequent formal request.

### Data collection

Interviews were developed based on issues identified in previous studies and related to professional role/setting; knowledge and awareness of RHD; and perspectives of health care provision for women with RHD-P (See Appendix 1) [3,15,24]. The 60–90 minute interviews were conducted by the lead author as part of a doctorate and her work in bi-national research investigating the impact of RHD-P [3,7,15]. They were held in person or by phone; taped, transcribed and imported into (password-protected) NVivo^©^ analysis software.

### Analysis

Thematic analysis of interviews identified meanings across and within transcripts and the overarching design that united them to address research questions [34]. Data familiarisation, data coding and theme development were conducted as a recursive process. Anticipated codes drawn from previous studies and a health systems lens informed the initial coding [3,15]. Interview audio/transcript reviews were supported by NVivo query reports. The coding process was carried out individually and in a workshop format to confirm member agreement. Strategies for rigor pursued during the study included credibility (member checking, peer review), transferability (context descriptions aimed to hold resonance with possible applicability in similar settings and provide a base for further research); dependability (providing a systematically documented framework); and confirmability (reference to other studies and supervisory discussions) [35].

## Results

Participants’ work bridged clinical and non-clinical care domains – maternity, cardiac and other specialisations; primary health; Aboriginal health-specific services, as well as RHD-specific strategies and programs. They worked in urban, regional and remote settings across four Australian jurisdictions (Figure 1, Tables 1 and 2). Nineteen participants were interviewed between 2014 to 2018, when data saturation was met.

**Table 1:** Participants – clinical and professional categories.


Midwife/Nurse	6
Obstetrician	3
Aboriginal Health Practitioner	1
Cardiologist	1
Physician	1
Anaesthetist	1
RHD program staff*	4


* The RHD Australia program supports the prevention, diagnosis and management of acute rheumatic fever (ARF) and RHD in Australia. It is funded under the Australian Government’s Rheumatic Fever Strategy.

**Table 2:** Participant attributes and characteristics – sector, location, organisation.


**Jurisdiction**	Northern Territory	8
	New South Wales	4
	Queensland	5
	Western Australia	2
**Location**	Regional with remote outreach	4
	Regional	8
	Urban with remote outreach	2
	Urban	5
**Workplace**	Hospital-based maternity	5
	Aboriginal Mothers and Babies service	3
	Aboriginal medical service or primary health	4
	Hospital – cardiac or high-risk specialist	3
	RHD Control program or strategy	4


The above tables do not adequately describe the professional and cultural backgrounds of participants, whose experience brought substantial breadth and depth. Their professional trajectories and expertise intersected Indigenous health, RHD advocacy and policy, obstetrics, midwifery and nursing, and other specialisations such as anaesthetics and cardiology, with all participants describing 15 or more years’ experience. Seven participants had worked in remote Australian settings for extended periods and were now working in urban locations. Four had long-term Aboriginal community experience and were currently working in hospital settings – or vice versa. Three participants had worked in other countries with a high prevalence of RHD.

### Themes

**Figure 2:**
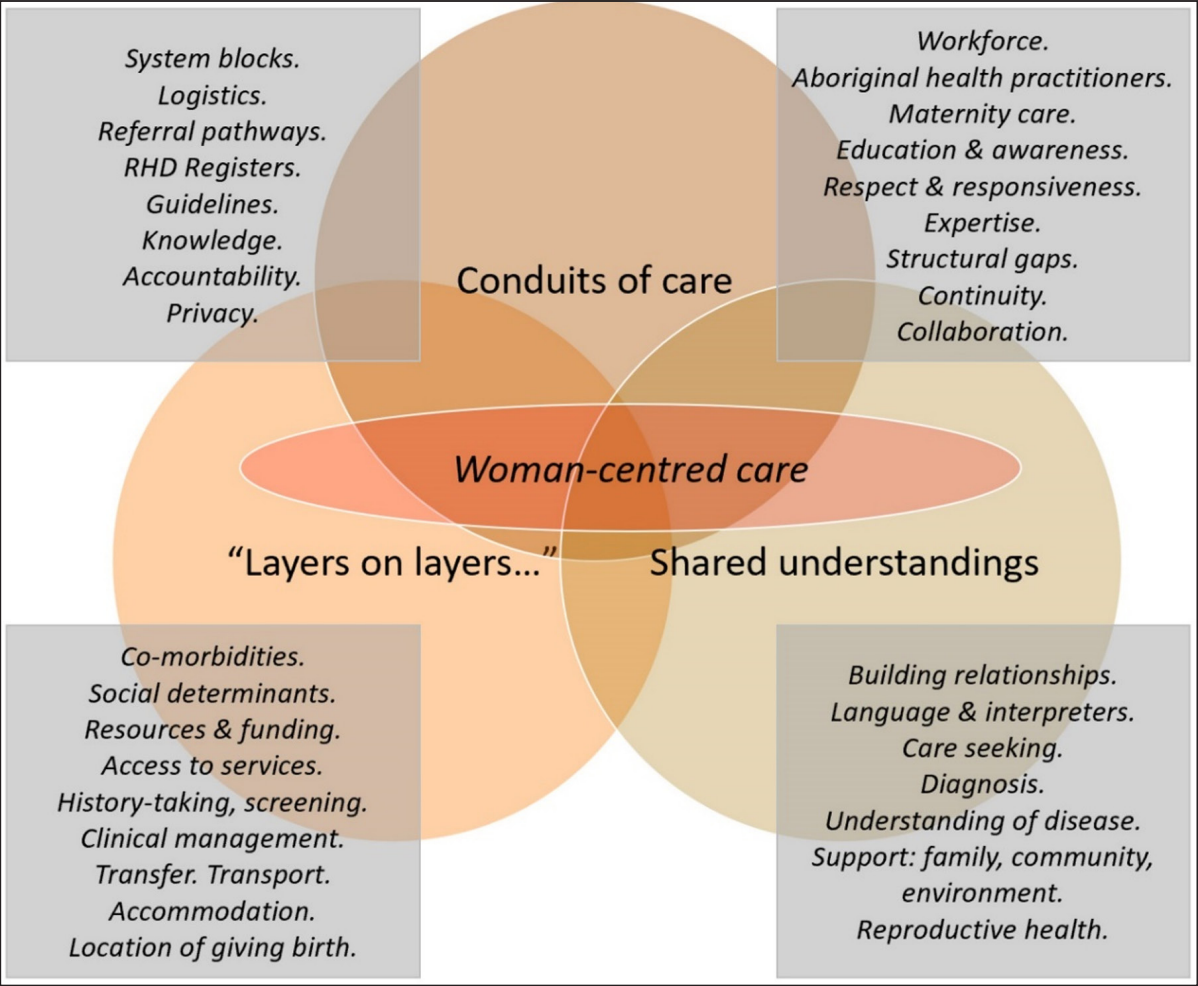
Themes related to care trajectories for women with RHD-P.

Three main themes were identified (Figure 2 above): Conduits of care; Shared understandings; and ‘Layers on layers,’ together with interconnected sub-themes.

### Conduits of care

Health system challenges relating to the workforce, access to services and a lack of both clinical and cultural awareness were described that diminished the capacity to provide collaborative woman-centred models of care for women with RHD-P. Silos related to processes, knowledge and clinical care were identified that fragmented the provision of health services.

#### System blocks

Participants expressed frustration in relation to managing information. They described patient referrals to specialist services (for instance, echocardiograms) that were not actioned and clinical test results not received. Multiple tiers of information systems across and between sectors (primary health, specialist and tertiary hospital services straddling multiple jurisdictions) and technologies (electronic health records and paper-based systems) were identified. Participants described accessing up to seven patient record databases, including two primary health systems and legacy paper-based systems to elicit patient history. These layers of information were reported to affect women’s diagnosis, including where a previously confirmed diagnosis of RHD was often ‘missed’ in a subsequent pregnancy.

These disconnects were more pronounced given women’s inter-community mobility, particularly in remote Australia. Women faced a number of issues accessing clinical services:

*Restrictions impact on women travelling for appointments. [Most] women have children to care for…. if they’re still breastfeeding but the child is over a certain age, the child isn’t funded to travel. We need more flexible and compassionate patient travel systems* [CL03].

Participants described women’s complex referral pathways that were subject to regulatory, logistical and geographical access constraints. For example, a woman who lived in one community may visit up to five hospitals across three jurisdictions that covered a distance of over 2000 km throughout her pregnancy:

*So, midwifery care is in one jurisdiction, the [cardiac and obstetric] tests and birth are in another – 1500 km apart. They’ll go to Townsville if they’re not well or Brisbane [2000 km], depending on severity. Or, if they’re in Alice Springs and get complications, they’d probably go to Adelaide [1500 km]* [CL011].

One midwife considered that care pathways for women with RHD in her Aboriginal Mothers and Babies (AMB) service generally worked well, noting that this was dependent on early recognition and a responsive referral system.

#### Workforce factors

Roles were discussed in the context of work structures that supported woman-centred care (or not), particularly in relation to Aboriginal health workers (AHWs) and practitioners (AHW&P)*. Participants expressed frustration when AHW&P skills were not recognised and opportunities to work collaboratively were missed:

*… when we don’t have [AHW&P], we don’t work well. ‘Cause they’re our connection, that cultural connection, you know, worth their weight in gold and I don’t think it’s appreciated how valuable their role is*. [CL03 Clinician working in urban tertiary centre and remote settings]

Aboriginal health worker and practitioner skills were not always fully optimised. One participant recounted where, on a busy clinic day, clients waiting for a secondary prophylaxis injection became frustrated and left whilst the senior Aboriginal health practitioner drove clients around, describing this as a ‘waste of talent.’ She continued that her role entailed *‘being expert in pulling everything together and doing that juggling’* (of meeting client and health service needs) [CL13].

Several participants commented on workforce shortages and skill gaps and the impact that had on the quality of care in remote Aboriginal communities that were so disproportionately impacted by RHD:

*If you want to work in Antarctica you have psychological screening and two years’ training. But if you want work [in remote Australia] looking after our most vulnerable people, you just go… And you don’t go through a vigorous selection process ‘cause they’re desperate for staff* [CR01].

* Aboriginal Health Worker: Includes non-clinical roles of Aboriginal Community Health Workers, Aboriginal Hospital Liaison Officer and Aboriginal Liaison Officer. Aboriginal Health Practitioner: provides direct clinical services to local Aboriginal communities.

#### Breaking down silos

Workarounds and initiatives were described to achieve more effective care. RHD program staff referred to ‘joining dots’ to ensure information was not missed. They asked the maternity service to highlight RHD-related information in women’s antenatal chart to circumvent the unwieldy health information systems described above.

The various AMB and RHD programs were often seen as a system-based ‘glue’ to harmonise services, particularly when based on a partnership model of AHW&Ps and (Indigenous or non-Indigenous) midwives or coordinators. This helped support the nexus between women, community and health services, to ensure improvements in antenatal and cardiac care.

*Often it will be the older females in the family – Mum, Aunty – who let health workers know when someone’s expecting and get them in for the tests…And then the women themselves start volunteering and asking questions. Then we’ll either link them in with the maternity services or they’ll come up themselves*. [SP04 RHD program coordinator]

A participant from a tertiary clinic described the brokering role they played in matching high-risk women’s preferences to those of the multidisciplinary team:

*So, the doctors might say, “This is what we need,” and I’ll say, “Well this is what she wants.” And then we try and make a care plan that fits in with both* [CL12].

Some workarounds required political strategising. One participant explained how they discretely worked ‘under the radar’ to optimise required care. These responses were often mediated by individual relationships, ingenuity and serendipity.

### ‘Layers on layers’

The complexities of addressing a chronic disease during pregnancy were described in relation to the social determinants of health. A backdrop of constraints included access to healthy housing, food security, and social barriers.

Competing priorities with co-morbidities such as diabetes and renal disease meant that RHD was often not ‘on the radar.’ A midwife in a high-risk antenatal clinic described RHD as being a drop in the ocean compared to other chronic diseases: *‘We’re just overwhelmed with obesity and diabetes’* [CL12].

Targeting RHD within these complex landscapes remained paramount for one participant who commented on the importance of addressing the ‘causes of the causes’ that underpinned so many other conditions:

*The thing about RHD is that it’s a sentinel disease for poverty… If you’re holding up RHD as being the poster child for poor living conditions, and you’re making efforts to improve those, it spills into improving [other] health conditions [CR01]*.

### Shared understandings: health literacy, awareness and education

Perspectives on health literacy regarding RHD-P varied. Participants described women not receiving information in a way that made sense for them. One spoke of having little focus on health education: *‘I can’t ever remember discussing RHD with a woman. Isn’t that terrible*.’ [CL11, maternity unit in a high RHD prevalence region].

The need to draw on multiple methods of providing information that made sense for women was described. One clinician spoke about the scaffolded approach they took when a heart murmur was noted: making the singing noise of the murmur. Pictorial content was preferred to text. The visual reference of antenatal ultrasonography – which many women were familiar with – also helped describe echocardiograms. Having conversations that promoted informed decisions on the part of women paralleled other health conditions:

*Of course, it’s not just RHD, it’s across other diseases. What’s the evidence-base, the best care for that person? Getting that woman to explain what they think is wrong with them and what they want to do. … So that they can support her in that process*. [CL08]

Providing information as distinct from sharing understandings was described:

*There are assumptions made along the lines of…*, “I have explained this.” *Well, we may have said the words. But what those words mean, in relation to that person are different things. We’re travelling along parallel pathways* [CL06].

The clinician continued to argue that health education was inappropriately over-simplified, rather than supporting conversations with women in a way that met their needs in the contexts of lives and culture.

Participants referred to health education films in Aboriginal languages that explore fertility, pregnancy and family for Indigenous women with RHD [36]. The significance of these Aboriginal and non-Indigenous co-designed and produced films was noted by one participant: ‘*…and we can see a young person’s eyes light up as soon as they see it’s in Language.’* [SP01]

Improved guidelines were called for, with one clinician expressing frustration at a lack of clarity to guide decisions in pregnancy.

One midwife in a regional AMB described the improved awareness for her AMB team through researcher collaboration and how this promoted strengthened knowledge and advocacy in conversations with other health services.

#### Trust

Descriptions of effective models of care were based upon shared respect between the health team, women and community members. An RHD coordinator illustrated how this trust was built slowly over time.

*We [AHW and RHD coordinator] both do a risk assessment in the home now. I got cheeky once and asked if I could bring a student. And Deb just looked at me, saying, “Sara, it’s taken me long enough to get them to let* you *come to their home.”* [SP03]

The importance of time was referred to by several participants: mostly in relation to the time taken to build confidence and trust in health providers (which in turn was premised on continuity of staff and respectful care), but also in taking time to sit with women which required ‘being brave’ and juggling long client lists against often complex clinical and social needs [CL06]. Time was also referred to in understanding that shifts in models of care were not quick-fix processes: *“Long term, it’s much better because we won’t get the chronic diseases in 20 years. But it’s a slow journey.”* [CL08]

#### Reproductive health

Participants spoke of reproductive health in the context of women’s individual circumstances. One AMB midwife described how they worked with women in a holistic way to help women understand the impact of choices of having a baby and inter-pregnancy spacing. Frustration was expressed where women presented without inter-pregnancy cardiac (or other required) follow ups. *‘… and so then you’re deciding if they need valvuloplasty during pregnancy.’* [CL09]

## Discussion

### Main findings

This qualitative study identified a lack of integrated women-centred health care during pregnancy for women with RHD in Australia from the perspective of health services. Findings highlight the need for a life-course approach that supports transition to adulthood, considers clinical interventions in the context of reproductive health and preconception care, and culturally safe pregnancy and postpartum care. These issues have global relevance, particularly in other populations where underlying determinants result in a high prevalence of RHD.

**Figure 3:**
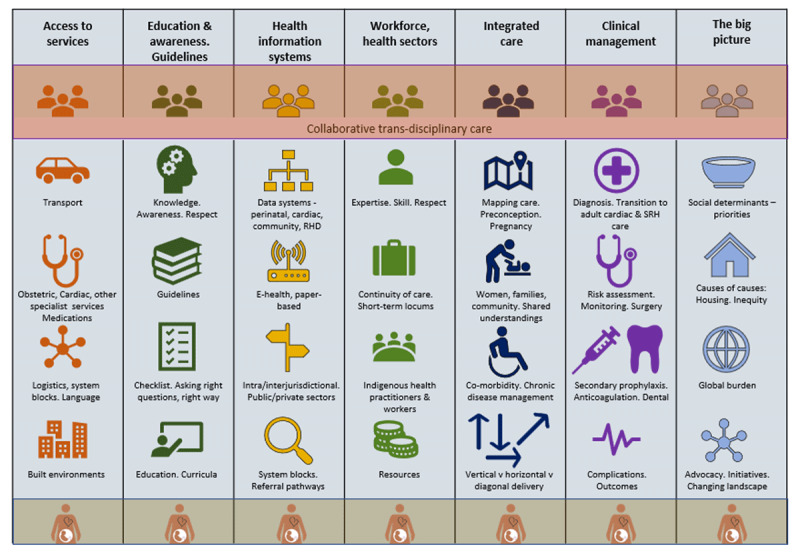
Perspectives of health services: what matters for pregnant women with RHD.

Three key themes emerged related to models of care: system blocks and conduits; layered complexities; and understandings of RHD care management. Underpinning these, a complex interplay (Figure 3) of systemic and cultural sub-themes were revealed.

Attributes of effective models of care were described including the importance of early diagnosis of RHD, access to services, respectful maternity and RHD care delivered by a skilled, collaborative workforce. These were associated with a mix of system-based and personnel-driven criteria, as listed in Figure 3 and described in more detail below.

Regulatory, logistical and access constraints impinged on the delivery of appropriate woman-centred care. A more compassionate approach to access including transport and accommodation support was called for.

Improved guidelines were called for to inform decision-making, particularly in relation to risk assessment and clinical interventions. Through the course of this research, shifts in Australian RHD policy and strategic approaches continued to evolve, particularly in relation to Guideline development [37] and education [38]. While there was an overall high awareness and knowledge by participants regarding RHD-P, they described an often-poor understanding more generally in maternity services. Other research of clinician awareness related to all-cardiac disease in women in high-income settings highlights similar deficits [39,40]. However, RHD carries added risk in the not-uncommon situation where previously undiagnosed disease becomes symptomatic during pregnancy or postpartum, in contrast to congenital heart disease.

The need to improve data systems and communications was a recurring theme. Disjointed systems in maternity, cardiac, Aboriginal and primary health care in particular, contributed to poor communications and challenged integrated care. Aspects of the systemic and structural impediments identified that continue to impede the redressing of Indigenous health injustices were consistent with a study examining Australian RHD practitioners’ perspectives in improvement of RHD prevention and care [41].

In our study, participants’ illustrations of compromised care pathways often centred on the workforce. Particularly in remote high-prevalence regions, participants pointed to an often short-term and locum workforce that precluded continuity of care.

Examples of quality care pathways in our study were embedded in an integrative approach that promoted improved clinical management, including early diagnosis, effective transition to adult cardiac care, risk management and interventions. Aboriginal Mothers and Babies health services were described as providing conduits of care that supported better outcomes [42,43,44,45,46,47]. So, too, RHD programs were referred to as providing these links.

Many of the issues described in this study are endemic in Australian (and other) health contexts. Gaps related to pre-conception care (cardiac and reproductive), coordinated multidisciplinary approaches, access to services and post-partum follow-up are consistent with other studies and reviews of all-cardiac disease in pregnancy in high income settings [48,49,50]. A metasynthesis of qualitative research investigating experiences of pregnant women with existing or acquired cardiac disease in high-income settings highlighted concerns regarding health information needs, and the need for women-centred care [51].

However, the underlying social determinants that give rise to RHD add dimensions that profoundly impact on women’s experiences of pregnancy, their health and lives. As a sentinel disease of poverty and health inequality, the persistence of this disease represents a systemic failure in addressing underlying these determinants of health.

### Parallels with other settings

Many aspects of the findings of our research are shared by other countries challenged by low-resources, poor access to services compounded by remoteness and lack of access to specialist services and gaps in health literacy among healthcare providers [18,52,53,54,55,56,57].

Much of the work on the impact of RHD in pregnancy that has informed subsequent research and guided policy has been conducted in sub-Saharan Africa [17,18,19,20,21,52,58,59,60,61]. More recent studies from Uganda in particular call for improved family/societal education programs and community engagement, leading to better outcomes and patient empowerment [62,63]. Pregnancy provides the opportunity to provide a women-centred, whole-of-life approach to this chronic condition, to improve cardiovascular and perinatal outcomes. While there were no initiatives described that specifically addressed the needs of pregnant women with RHD in Australia, findings from our study suggests that health services can learn from other programs that support the provision of improved care.

Maternity care partnership models – where Aboriginal health practitioners and (Indigenous and non-Indigenous) midwives work together with other health services to provide care during pregnancy and the early post-partum – have consistently demonstrated improved outcomes [43,44,64,65]. These models draw on Birthing on Country principles, described as *‘…a metaphor for the best start in life for Aboriginal and Torres Strait Islander babies and their families’* which provide an integrated, holistic and culturally appropriate model of care [66]. Such principles are congruent with many of the strategies called for or described by participants in this study.

Similarly, fundamental drivers of successful cardiac care programs for Indigenous Australians have incorporated engagement, recovery interventions, capacity building and self-governance, with a small number of services that have addressed all or part of these [67,68,69,70,71].

Despite often complex care requirements, pregnancy for women with RHD provides a unique opportunity to strengthen health system responses, improve care pathways, address whole-of-life health and ultimately reduce the burden of RHD for women. To respond effectively, structural and cultural changes are required to improve health system agility and capability. This includes enhanced investment in education and capacity building – particularly in maternal health – to support a better informed and skilled workforce; promote shared understandings, improve information systems and reporting of core indicators to more accurately benchmark respectful care pathways, outcomes and burden of RHD-P.

This approach demands an integrated health care response from a motivated and skilled health workforce.

### Strengths and limitations

This paper provides insight into factors impacting on the continuum of respectful, appropriate care for women with RHD in pregnancy, identifying barriers and enablers of quality models. The authors are not aware of any equivalent study in other high-income settings where the burden of RHD is so disproportionately high for Indigenous women.

There were potential limitations identified. Interviews were single events only. It could be argued that a more prolonged engagement would minimise the risk of participant bias [72]. Member checking was conducted to verify and clarify statements where required. Additionally, two other studies undertaken served to broaden perspective and reinforce findings [7,24].

There was considerable participant heterogeneity in professional, cultural and language characteristics and findings should not be considered to represent specific professional views. The transferability of findings to other settings and contexts is a consideration for this study. Aboriginal and/or Torres Strait Islander women represent nearly 80% of the burden of RHD-P in Australia [3]. Precepts of woman-centred care may vary for other populations, such as Maori or Pasifika women, refugees, or other immigrant women. Such variation equally applies in other country settings. However, broad principles related to optimal care pathways for women with RHD may be applicable in other contexts, with aspects of findings and discussion on models of care corroborated by other studies in low-resource settings that include women with RHD [21,60,62].

Interviews were held over a four-year period. During this time the RHD political, policy and funding landscape evolved significantly [14,73]. Advocacy and education resource initiatives are being increasingly informed and directed by the lived experience of those with RHD [74].

## Conclusion

A complex interplay of systemic and cultural factors impacts the provision of optimal health care for women with RHD-P. Emerging from the study findings was a recognition that optimal care requires a woman-centred life-course approach that supports transition to adult care, considers surgery and other interventions in the context of reproductive health and preconception care, as well as pregnancy and postpartum care.

Central to the provision of informed, respectful care for women with RHD is timely diagnosis, access to health services and continuity of care. The research recommendations based on study findings aim to better achieve these goals to ensure the needs of women with RHD are better met. Underpinning these findings is the need for partnership models that support integrated care for women and girls with RHD throughout the life-course.

For key goals to be met in the context of RHD [2], maternal health must be better integrated into RHD strategies and RHD must be better addressed in maternal health [75,76,77].

## Additional File

The additional file for this article can be found as follows:

10.5334/gh.1086.s1 Appendix 1. Research questions and interview question guide.
