# Supplementary material for: Caring for Pregnant Women with Rheumatic Heart Disease: A Qualitative Study of Health Service Provider Perspectives
Source: Glob Heart. 2021 Dec 22;16(1):88. doi: 10.5334/gh.1086 (PMC8698228; doi:10.5334/gh.1086)
Supplement: Appendix 1. — Research questions and interview question guide. [file gh-16-1-1086-s1.pdf]

Appendix 1: Research questions and Interview guide

Caring for pregnant women with rheumatic heart disease: A qualitative study of health service provider perspectives

Research questions

1. What degree of knowledge, expertise and awareness exists amongst health professionals providing care for women with RHD in pregnancy?
2. What needs do Aboriginal and non-Aboriginal women have that are not currently addressed in health service access, counselling and clinical management of RHD in pregnancy, and how does that vary between services?
3. What barriers exist to the provision of optimal health care from the perspectives of health professionals who provide that care?
4. How can health services more effectively meet the needs of these women, including access, education, counselling and clinical management of RHD in pregnancy?
5. What factors enhance/hinder the capacity of health information systems (medical record systems, RHD Control Program registers) to support health professionals in providing timely well-informed care for pregnant women with RHD?

Topics

T1 Professional role and location (maternity, cardiac, Aboriginal health, remote health, RHD Control program staff).

T2 Knowledge and awareness of ARF/RHD: disease, treatment, guidelines, management (general and in pregnancy). Knowledge of access to resources.

T3 Experience of provision of care for pregnant women with RHD: when diagnosed, what the woman was told, her knowledge and understanding of the disease and treatment. Health professionals' experience and understanding of secondary prophylaxis, echocardiography, issues about anticoagulation. Experience and understanding of surgical or other interventions.

T4 Health care service provision for pregnant women with RHD: logistics, access, strategies, policies, resources. Challenges and gaps. Identifying what works and what doesn't.

T5 Urban and regional/remote differences in health (general, cardiac and maternity) care access and services.

T6 Health information systems and surveillance: what degree of coordination exists between systems to support optimal and timely sharing of information? What gaps exist? How integral is the RHD Control register perceived as being in the provision of care for women with RHD? What gaps exist, and what strategies have been successful in promoting timely effective sharing of health information?

Interview guides

- Clinical
- Non-clinical

Clinical

Conversational style in your own words at appropriate times	Topic
1. **Professional and experience:**<br>***Tell me about your experience working with women with RHD.***	T1
Role; type of health unit (maternity – antenatal, labour ward, postnatal, MGP, remote health, cardiac); length of time and experience in this or other clinical setting.	T5
2. **Knowledge/awareness. Models of care**<br>***Can you describe the process of identification and clinical care for pregnant women with RHD in your centre/unit?***	T2<br>T3
Notification – health information systems, surveillance strategies	T4
Knowledge/awareness of management strategies - Secondary Prophylaxis (SP), safety in pregnancy	T5
Knowledge/awareness of ARF/RHD Register, how to access, which jurisdictions	T6
Use of resources - online course, phone app, guidelines, quick reference.	
Clinical policies/guidelines in this clinical setting associated with the care of pregnant women with RHD? Continuous Quality Improvement (CQI) or similar program?	
Resource impact - where does ARF/RHD sit in relation to other (chronic) diseases/conditions in this service/centre/unit (prevalence, resource impact, awareness)	
Changes in the approach to care for pregnant women with RHD over the last five/ten years?	

3. **Delivery of care:** T3

What are the key issues in delivery of care for women with RHD in pregnancy?

Collaborative care with cardiac, maternity, Aboriginal health, AMIC, services, RHD Control Register (Other?) T4

T5

Continuity of care. Logistics of coordination – antenatal and cardiac care – SP treatment, echo.

Health information systems, information transfer

Transfer, transport, access to services

Support for women - interpreter, Aunty, Aboriginal Health practitioner.

Disconnect between model of care policy and practice.

4. Education and health literacy for women: T3

What do you ask/talk about with pregnant women who have RHD? How would you describe women's understanding of the pathology of the disease? Examples? Resources you use? What would support Improved understanding/adherence to treatment? T4

Heart pathophysiology, SP and other preventive strategies, management, anticoagulation if relevant, contraceptive counselling, RHD in pregnancy, adherence to treatment.

Who attends – partner, interpreter, Aunty, maternity, cardiac.

5. Service structures T6

What changes could be made to better meet the needs of pregnant women with RHD? T5

Provision and structure of services – cardiac, Aboriginal health, remote.

Access - transport, transfer

Health information systems – data collected and how this is managed/shared. Efficiencies and accuracy.

6. **Experiences** T3

Thinking about your experiences of care for women with RHD in pregnancy. Can you remember a time where you thought things went well for her care. Why? What happened? Can you remember a time where you thought there were challenges in her care. Why? What happened? T4

7. **Other comments/thoughts?**

Non-Clinical

Conversational style in your own words at appropriate times **Topic**

1. **Professional and experience:** T1

Tell me about your experience working with women with RHD. T5

Role; type of service/facility/function; any experience in clinical settings.

1. **Knowledge/awareness. Models of care** T2

Can you describe what you know about the process of identification of RHD and how that's managed in pregnancy. Resources available. T3

Notification – health information systems, surveillance strategies T4

Knowledge/awareness of management strategies - Secondary Prophylaxis (SP), safety in pregnancy T5

T6

Knowledge/awareness of ARF/RHD Register, how to access, which jurisdictions

Use of resources - online course, phone app, guidelines, quick reference.

Clinical policies/guidelines associated with the care of pregnant women with RHD? Continuous Quality Improvement (CQI) or similar program?

Resource impact - where does ARF/RHD sit in relation to other (chronic) diseases/conditions in this setting (prevalence, resource impact, awareness, policy priority)

Changes in the approach to care for pregnant women with RHD over the last five/ten

years?

2. **Delivery of care:** T3
What are the key issues in delivery of care for women with RHD in pregnancy?

Collaborative care with cardiac, maternity, Aboriginal health, AMIC, services, RHD Control Register (Other?) T4 T5

Continuity of care. Logistics of coordination – antenatal and cardiac care – SP treatment, echo.

Health information systems, information transfer

Transfer, transport, access to services

Support for women - interpreter, Aunty, Aboriginal Health practitioner.

Disconnect between model of care policy and practice.

3. Education and health literacy for women: T3
What resources are available for women with RHD? What could be done to improve understanding/awareness/knowledge of the disease (particularly in pregnancy) for women? For those working in clinical settings? T4

Health literacy: for women; for those working in clinical settings.

Resources and availability

8. **Service structures** T6
What changes could be made to better meet the needs of pregnant women with RHD? What are the key issues in delivery of care? T5

Provision and structure of services – cardiac, Aboriginal health, remote.

Access - transport, transfer

Health information systems – data collected and how this is managed/shared. Efficiencies and accuracy.

9. Other comments/thoughts?
